# Variation among cleft centres in the use of secondary surgery for children with cleft palate: a retrospective cohort study

**DOI:** 10.1136/bmjpo-2017-000063

**Published:** 2017-08-31

**Authors:** Thomas J Sitzman, Monir Hossain, Adam C Carle, Pamela C Heaton, Maria T Britto

**Affiliations:** 1 Division of Plastic Surgery, Cincinnati Children's Hospital Medical Center, Cincinnati, Ohio, USA; 2 James M. Anderson for Health Systems Excellence, Cincinnati Children's Hospital Medical Center, Cincinnati, Ohio, USA; 3 Division of Biostatistics and Epidemiology, Cincinnati Children's Hospital Medical Center, Cincinnati, Ohio, USA; 4 James L. Winkle College of Pharmacy, University of Cincinnati Academic Health Center, Cincinnati, Ohio, USA; 5 Division of Adolescent and Transition Medicine, Cincinnati Children’s Hospital Medical Center, Cincinnati, Ohio, USA

**Keywords:** health services research, outcomes research, plastic surgery, procedures

## Abstract

**Objectives:**

To test whether cleft centres vary in their use of secondary cleft palate surgery, also known as revision palate surgery, and if so to identify modifiable hospital factors and surgeon factors that are associated with use of secondary surgery.

**Design:**

Retrospective cohort study.

**Setting:**

Forty-three paediatric hospitals across the USA.

**Patients:**

Children with cleft lip and palate who underwent primary cleft palate repair from 1999 to 2013.

**Main outcome measures:**

Time from primary cleft palate repair to secondary palate surgery.

**Results:**

We identified 4939 children who underwent primary cleft palate repair. At 10 years after primary palate repair, 44% of children had undergone secondary palate surgery. Significant variation existed among hospitals (p<0.001); the proportion of children undergoing secondary surgery by 10 years ranged from 9% to 77% across hospitals. After adjusting for patient demographics, primary palate repair before 9 months of age was associated with an increased hazard of secondary palate surgery (initial HR 6.74, 95% CI 5.30 to 8.73). Postoperative antibiotics, surgeon procedure volume and hospital procedure volume were not associated with time to secondary surgery (p>0.05). Of the outcome variation attributable to hospitals and surgeons, between-hospital differences accounted for 59% (p<0.001), while between-surgeon differences accounted for 41% (p<0.001).

**Conclusions:**

Substantial variation in the hazard of secondary palate surgery exists depending on a child’s age at primary palate repair and the hospital and surgeon performing their repair. Performing primary palate repair before 9 months of age substantially increases the hazard of secondary surgery. Further research is needed to identify other factors contributing to variation in palate surgery outcomes among hospitals and surgeons.

**What is already known on this topic?**

- Treatment approaches for children with cleft palate differ broadly based on the cleft centre and cleft surgeon delivering care.
- The effect of these different approaches on clinical outcomes, and in particular the need for a secondary palate surgery, is unclear.

**What this study hopes to add?**

- Among children with cleft palate, almost half will undergo secondary palate surgery. However, there is greater than four-fold variation among centres in their use of secondary surgery.
- These results suggest substantial opportunity for cleft centres and surgeons to improve outcomes for children with cleft palate by reducing variation in treatment approach.

## Introduction

Cleft palate occurs in approximately 1 of every 1000 births, making it one of the world’s most common congenital anomalies.^1–3^ Children undergo cleft palate repair early in life, with the goals of closing the abnormal connection between the mouth and nose and obtaining normal speech. Failure to achieve either goal substantially impacts a child’s quality of life by leaving a fistula that allows liquids and food to exit their nose while eating or by making their speech unintelligible.^4^ Either complication may lead children to undergo secondary palate surgery. Secondary surgery requires a major operation, several weeks recovery and medical costs similar to the primary repair.^5^

Patients with cleft palate are principally cared for in cleft centres, which are multidisciplinary teams typically associated with a single hospital and multiple cleft surgeons. Cleft centres exhibit broad variation in both their treatment approach^6^ and their complication rates after palate repair. Fistula incidence ranges from 0% to 35%.^7^ Speech problems attributable to abnormal palate function range from 10% to 56%.^4 8 9^ This variation in both fistulae and speech problems may lead to differences in rates of secondary palate surgery.

Understanding the variation in secondary surgery along with causative factors for this variation may impact how, where and by whom treatment is delivered. There are many controversial elements of cleft palate care, including use of postoperative antibiotics^10^ and the importance of hospital volume and provider volume.^11 12^ Timing of primary cleft palate closure is also controversial: some providers advocate performing repair before 9 months of age to improve speech outcomes,^13–15^ while others delay palate repair until 12 or 18 months of age when the palate has grown larger and the risk of harming future facial growth may be reduced.^16 17^

Previous comparisons of secondary palate surgery among cleft centres in England, Denmark, Sweden and the Netherlands identified significant differences in outcomes among centres, and these findings led to government-mandated changes in cleft surgery delivery.^18^ A study of four centres in the USA and Canada found the hazard of secondary palate surgery varied sixfold across teams, although these differences were not statistically significant (95% CI 0.76 to 45.65, p=0.057).^19^ Thus, it remains unknown whether variability in secondary palate surgery seen in Western Europe generalises to cleft centres around the globe.

The present study is a large, retrospective analysis of children undergoing cleft palate repair at 43 free-standing children’s hospitals in the USA. We tested our hypotheses that (1) significant variation exists among hospitals in the use of secondary palate surgery and (2) modifiable hospital factors and surgeon factors at the time of primary palate repair, including age at primary palate repair, hospital volume and surgeon volume, use of postoperative antibiotics and duration of postoperative hospitalisation, are associated with subsequent secondary palate surgery.

## Patients and methods

### Study design and data source

We performed a retrospective cohort study of children with cleft lip and palate who underwent cleft palate repair at children’s hospitals in the USA contributing to the Pediatric Health Information System (PHIS). PHIS contains detailed administrative data for all inpatient admissions at participating hospitals. Extensive processes ensure data quality and reliability.^20^ PHIS contains a hospital-specific patient identifier that enables tracking a patient across all admissions at an individual hospital. Forty-three free-standing children’s hospitals participate in PHIS, accounting for 85% of children’s hospital admissions in the USA.^21^

The Cincinnati Children’s Hospital Medical Center Institutional Review Board reviewed this study and determined it was not human subjects research, as defined by the Common Rule (45CFR46.102[f]), because the dataset was deidentified.

### Study population

Children younger than 2 years diagnosed with cleft lip and palate who were discharged between 1 January 1999 and 30 December 2013, after having undergone cleft palate repair were included. Patients with complex chronic conditions that might influence treatment of their cleft palate, including 22q11.2 deletion syndrome, were excluded using previously validated *International Classification of Diseases, Ninth Revision (ICD-9) codes*.^22^ Standard care for cleft palate repair includes palate repair before 2 years of age, so patients with initial palate repair after this age were excluded as they may possess additional medical conditions influencing palate repair timing. If a child underwent cleft palate repair at an individual hospital more than once during the study period, only the earliest repair was included; subsequent repairs were considered secondary palate surgeries.

### Study definitions

Study patients were identified using the *ICD-9* codes indicating cleft lip and palate (749.20–749.25) in any discharge diagnosis field. Among these patients, admissions during which the patient underwent primary palate repair were determined by the presence of the *ICD-9* code for correction of cleft palate (27.62). Any subsequent patient encounter at the same hospital that included an *ICD-9* code for correction of cleft palate (27.62), revision of cleft palate repair (27.63) or other plastic repair of palate (27.69) was defined as a secondary palate surgery. No information was available on receipt of secondary palate surgery at hospitals other than the patient’s initial treating facility.

### Outcome

The outcome variable was time from primary palate repair until the patient underwent secondary palate surgery. Patients not undergoing secondary surgery during the observation period were censored on 30 December 2013, the last date for which outcome status was available.

### Covariates

Covariates were defined using information available in PHIS from the patient’s primary palate repair. Demographic data included sex, race and median household income by postcode of residence. Race was included in the final model because prior research suggests variation among racial/ethnic groups in receipt of cleft palate surgery.^23 24^ Median household income by postcode of patient residence was obtained from 2010 US Census data and split into four categories based on US federal poverty guidelines for a family of four in 2010, as previously described.^25^

Additional covariates were specified a priori. Age at primary palate repair was categorised into three groups based on existing approaches to timing of primary palate repair: less than 9 months, 9–15 months and 16–24 months.^13 26 27^ Postoperative antibiotic use was defined as receipt of any antibiotic on the first and/or second day after primary palate repair and was dichotomised as present or absent. We dichotomised hospital length of stay as less than two nights and two or more nights to test the hypothesis that increased length of stay would reduce secondary surgery by enabling improved parent education on postsurgical care.

Surgeon and hospital procedure volume was determined for each patient on the day of the patient’s primary palate repair by counting all cleft palate repairs (*ICD-9* codes 27.62 and 27.63) performed by that surgeon or hospital, respectively, in the prior 365 days. This approach decreases exposure misclassification compared with annual procedure volume for the same year the procedure was performed.^28^ Specifically, procedure volume was used as a measure of experience; any procedures performed after a specific patient’s procedure do not contribute to the surgeon’s or hospital’s experience at the time that patient’s procedure was performed. Thus, annual procedure volume for the year the procedure was performed misclassifies experience, and the approach used here reduces misclassification by determining volume using the 365 days prior to each patient’s procedure.

Procedure volume counts included all cleft palate repairs in patients younger than 4 years of age, regardless of cleft type or additional medical conditions, as all palate repairs were predicted to increase the surgical team’s experience with the procedure. For surgeons or hospitals who had reported in PHIS for fewer than 365 days at the time of a patient’s palate repair, procedure volume was calculated as the number of cleft palate repairs performed during their first 365 days of reporting. Procedure volume was categorised into tertiles.

### Statistical analyses

We calculated descriptive statistics with means and percentages. We plotted a Kaplan-Meier time-to-event curve for all patients in the cohort.^29^ Prior to investigating variation in time-to-event among hospitals, we performed a power analysis to ensure we had a power of 0.8 to detect a 50% difference in time to secondary palate surgery among hospitals, with a type I error rate of 0.05, adjusted for pairwise comparisons.^30^ Assuming administrative censoring would occur and that secondary palate surgery would occur during the observation period for 47% of patients (from descriptive analysis of the data), we determined it was necessary to restrict this subgroup analysis to hospitals with at least 175 patients during the study period. We then plotted Kaplan-Meier time-to-event curves for hospitals meeting this criterion. We tested for variation in time-to-event among hospitals using a Log-rank test that stratified by patient gender, race and median household income by ZIP code of patient residence.

We then fit a three-level mixed-effects parametric survival-time model with clustering of patients within surgeons and clustering of surgeons within hospitals. We assumed a Weibull distribution for this model, after confirming appropriateness of this assumption by visual inspection of log–log plots of survival. Random effects were assumed to have normal distributions with zero means. We tested for all interactions. After fitting the full model, we estimated the variability attributable to the surgeon and hospital.

Sensitivity analyses were conducted to evaluate whether the choice of follow-up time imposed bias through right censoring. Sensitivity analyses included: (1) censoring children 2 years after their last encounter; (2) censoring children 4 years after their last encounter; (3) excluding children with less than 1 year of follow-up; and (4) excluding children with less than 4 years of follow-up. Results of all sensitivity analyses were nearly identical to those of our main analysis and are not presented. In a separate sensitivity analysis, we repeated our main analysis while adjusting for year of primary cleft palate repair; results were similar to our primary analysis and are included in online supplementary file 1.

10.1136/bmjpo-2017-000063.supp1 Supplementary file 1 supplementary file 2

Statistical analyses were performed using Stata V.13 and V.14 (StataCorp, College Station, Texas, USA). Statistical significance was set at p<0.05.

## Results

A total of 5846 children underwent cleft palate repair before 2 years of age at PHIS hospitals. We excluded 907 children with additional complex chronic conditions.^22^ Our final cohort included 4939 children who underwent primary cleft palate repair between 1999 and 2013. Secondary palate surgery was performed in 1421 children (29%) during the observation period; 71% of the observations (n=3518) were right-censored. Characteristics of the study population are shown in table 1.

**Table 1:** Characteristics of patients and the care delivered at their initial palate repair

Characteristic	No. (%)
Total	4939
Sex	
Male	3144 (64)
Female	1795 (36)
Race	
White	3374 (68)
Black	322 (7)
Asian or Pacific Islander	342 (7)
American Indian	66 (1)
Other	589 (12)
Not specified	246 (5)
Median annual household income of ZIP code	
$33 525 or less (<1.5 FPL^*^)	1230 (25)
$33 526–$44 700 (1.5–2 FPL)	1555 (32)
$44 701–$67 050 (2–3 FPL)	1527 (31)
$67 051 or more (>3 FPL)	483 (10)
No data available	144 (3)
Age at primary palate repair	
<9 months	1164 (24)
9–15 months	3186 (64)
16–24 months	589 (12)
Postoperative antibiotic use	
None	2537 (51)
Yes	2402 (49)
Surgeon procedure volume (on day of repair)	
Low (<10 repairs in prior year)	1307 (27)
Medium (10–25)	2343 (47)
High (>25)	1289 (26)
Hospital procedure volume (on day of repair)	
Low (<25 repairs in prior year)	1068 (22)
Medium (25–50)	2568 (52)
High (>50)	1303 (26)
Length of stay after surgery	
≤1 night	3034 (61)
≥2 nights	1905 (39)

*FPL, US Federal Poverty Level for a family of four.

**Figure 1:**
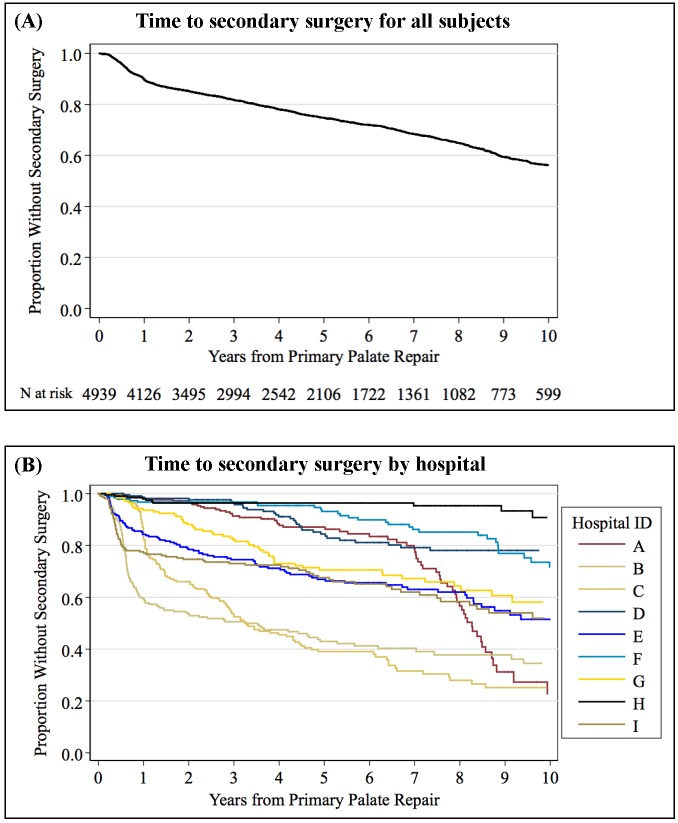
Kaplan-Meier curves for time until secondary palate surgery. (A) Time to secondary surgery for all patients in the study. (B) Time to secondary surgery by hospital for hospitals with >175 patients undergoing palate repair during the observation period. At-risk table and censoring times for each hospital are shown in figure 3 online supplementary file 3. Log-rank test is stratified by patient gender, race and median household income for ZIP code of residence.

Time to secondary surgery for the cohort is shown in figure 1A. At 3 years after primary palate repair, 18% of patients had undergone secondary palate surgery. At 5 years after primary palate repair, 25% of patients had undergone secondary palate surgery. At 10 years after primary palate repair, 44% of patients had undergone secondary palate surgery.

10.1136/bmjpo-2017-000063.supp3 Supplementary file 3 supplementary file 1

### Variation among hospitals

Figure 1B displays the variability in time to secondary surgery among the nine hospitals with at least 175 patients. Significant differences in time to secondary palate surgery existed across hospitals (p<0.001, stratified Log-rank test). At 10 years after primary palate repair, the proportion of children who had undergone secondary palate surgery ranged from 9% at hospital H to 77% at hospital A.

### Hospital-specific and surgeon-specific predictors

Using a mixed-effects time-to-event model, we investigated the patient and surgical factors associated with time to secondary surgery (table 2). This analysis included all PHIS hospitals. After adjustment for patient demographics (sex, race and household income level), postoperative antibiotic use, surgeon procedure volume, hospital procedure volume and length of stay after surgery were not associated with time to secondary surgery. Age at primary palate repair was associated with time to secondary palate surgery, with the hazard of secondary surgery increased for children who underwent primary repair before 9 months of age (p<0.001).

**Table 2:** Adjusted HRs for secondary palate surgery

Risk factor	Secondary palate surgery*	
	HR (95% CI)	p Value
Sex		0.70
Male	0.98 (0.87 to 1.10)	
Female	Reference	
Race		0.10
White	Reference	
Black	0.75 (0.58 to 0.97)	
Asian or Pacific Islander	1.01 (0.77 to 1.32)	
American Indian	1.09 (0.59 to 2.02)	
Other	0.97 (0.81 to 1.18)	
Not specified	1.28 (0.99 to 1.65)	
Median annual household income of ZIP code		0.16
$33 525 or less (<1.5 FPL†)	Reference	
$33 526–$44 700 (1.5–2 FPL)	0.95 (0.82 to 1.10)	
$44 701–$67 050 (2–3 FPL)	0.84 (0.72 to 0.98)	
$67 051 or more (>3 FPL)	0.91 (0.74 to 1.14)	
Age at primary palate repair		<0.001
<9 months‡		
At baseline	6.74 (5.20 to 8.73)	
At 1 year after repair	4.70 (3.44 to 6.43)	
At 5 years after repair	1.11 (0.66 to 1.89)	
9–15 months	1.15 (0.94 to 1.42)	
16–24 months	Reference	
Postoperative antibiotic use		0.06
None	Reference	
Yes	0.86 (0.74 to 1.01)	
Surgeon procedure volume (on day of repair)		0.17
Low (<10 repairs in prior year)	Reference	
Medium (10–25)	1.11 (0.94 to 1.31)	
High (>25)	1.25 (0.99 to 1.58)	
Hospital procedure volume (on day of repair)		0.14
Low (<25 repairs in prior year)	Reference	
Medium (25–50)	0.94 (0.77 to 1.14)	
High (>50)	0.78 (0.60 to 1.02)	
Length of stay after surgery		0.33
≤1 night	1.07 (0.94 to 1.22)	
≥2 nights	Reference	

Model assumes clustering of patients within surgeons and clustering of surgeons within hospitals; p<0.001 for likelihood-ratio tests of theta=0 for both surgeon and hospital.

†FPL, US Federal Poverty Level for a family of four.

‡Age less than 9 months at primary repair is a time varying covariate, with baseline HR 6.74 (5.20−8.73) that decreases by 30% (26–34%) each subsequent year.

**Figure 2:**
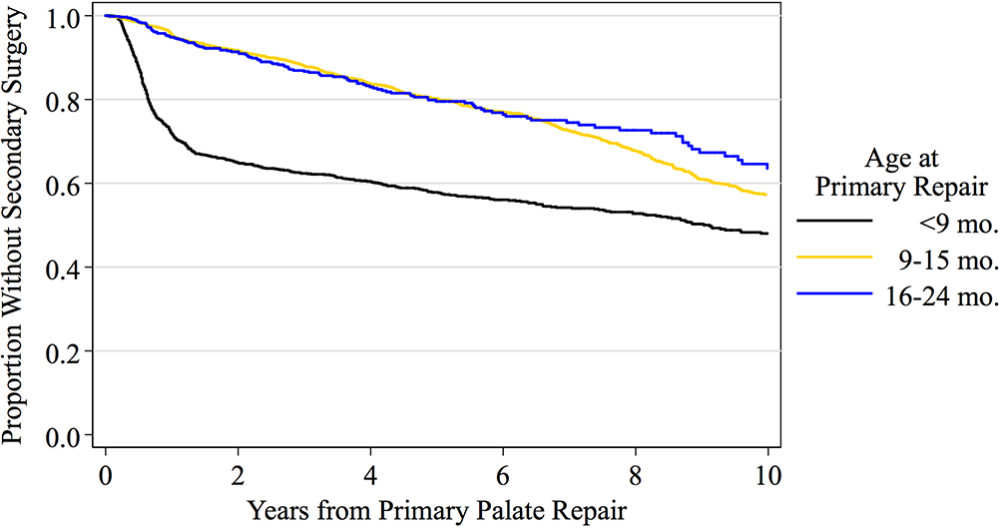
Kaplan-Meier curve for time until secondary surgery based on age at primary palate repair. This figure demonstrates the time-dependent hazard of secondary surgery. For children who underwent primary palate repair before 9 months of age, the hazard of secondary surgery lies principally in the first 2 years after primary repair.

As shown in figure 2, for children when underwent primary palate repair before 9 months of age, the hazard of secondary surgery was greatest immediately following the primary repair and diminished as the time since primary repair increased. Immediately following primary repair, children who had repair before 9 months of age had a 6.74-fold increased hazard of secondary surgery (95% CI 5.20 to 8.73) compared with children who underwent repair at 16–24 months of age. For children who did not undergo secondary repair during the first year after palate repair, their hazard of secondary surgery diminished slightly, with a HR of 4.70 (95% CI 3.44 to 6.43). For children who reached the fifth anniversary of their palate repair without undergoing secondary surgery, their hazard of secondary palate surgery at any time in the future was similar to children who had primary repair at 9–24 months of age. Among children who did not undergo secondary surgery, the duration of follow-up was greatest for children undergoing repair before 9 months of age (p<0.001, online supplementary file 2).

10.1136/bmjpo-2017-000063.supp2 Supplementary file 2 supplementary file 3

From the mixed-effects model, we estimated the variation attributable to hospitals and surgeons. Between-hospital differences accounted for 59% of this variation (p<0.001), while between-surgeon differences accounted for 41% (p<0.001).

## Discussion

We found substantial variability in secondary surgery for children with cleft palate treated at children’s hospitals in the USA. Ten years after primary palate repair, 44% of children underwent secondary surgery, but this varied from 9% to 77% among hospitals. Performing primary palate repair before 9 months of age was associated with a significantly increased hazard of secondary surgery. After adjusting for patient demographics, procedure volume, antibiotic use and timing of primary palate repair, the remaining variability in outcome was attributable to both between-hospital and between-surgeon differences. These results suggest there is a substantial opportunity for hospitals and surgeons to reduce the need for secondary palate surgery in children with cleft palate by reducing variation in treatment approach.

These findings are consistent with and extend prior reports by confirming that broad differences in secondary surgery among cleft centres in Western Europe also occur in the USA. During the 1990s, investigators found differences in both clinical outcomes and use of secondary surgery among cleft centres in Western Europe.^12 18 31–34^ Cleft centres in the UK achieved the worst outcomes, and this led them to consolidate centres and impose statutory mandates for outcome reporting from each centre.^35^ Small intercentre studies in the USA found similar differences in clinical outcomes and use of secondary surgery across cleft centres.^19 36–38^ However, our study is the first to examine whether differences among smaller groups of North American cleft centres generalise to cleft teams in the USA.

Although the best achievable rate of secondary surgery is not known and may vary among sites, the variation from 9% to 77% observed in this study is substantial. The literature does indicate that fistula rates below 10% are readily achievable, as are rates of secondary surgery for speech disorders below 20%.^7 39 40^ The results of this study suggest that many US hospitals achieve these results, and there is an opportunity to learn from these hospitals.

### Age at cleft palate repair

While there is universal agreement that ‘primary palatal repair should be done at the age that allows optimal speech development and facial growth’,^27^ there is little agreement about precisely what that age should be.^13–17 27^ Currently, the debate is between primary repair at 6 versus 12 months of age.^41^ Dorf and Curtin^13^ showed that children who undergo palate repair after 12 months of age exhibit more compensatory articulations, which require speech therapy to correct. This result has been confirmed by several others, yet none of these studies examined rates of secondary surgery for both speech and fistulae.^15 26^ Our results show the hazard of secondary surgery is initially 6.7-fold higher for children who have palate repair before 9 months of age and that this increased hazard persists for 5 years after surgery. We do not believe this increased hazard with early palate repair is due to underlying patient differences, as complicating medical or socioeconomic conditions lead to repair at older rather than younger ages in our experience. The Timing of Primary Surgery for Cleft Palate trial is an ongoing randomised control trial comparing palate repair at 6 and 12 months of age and results should be available by 2021. Until then, our study suggest that surgeons and hospitals should be cautious to recommend cleft palate repair before 9 months of age and do so only after reviewing their own results.

### The volume–outcome relationship

Our study failed to show an association between hospital volume and surgeon volume of cleft palate repairs and time until secondary palate surgery. A previous study of cleft palate surgery volume found surgeons who performed ≥3 palate repairs per year achieved superior speech outcomes to surgeons performing <3 repairs per year.^11^ While three repairs per year may be an important threshold, in the present study, only 6% of repairs were performed by surgeons below that threshold. We chose to set the threshold for low volume surgeons at <10 repairs per year and found that performing 10 or more palate repairs per year did not improve surgical outcomes. This is consistent with prior studies in paediatric surgery that suggested the effects of increased volume are dependent on the specific procedure, outcome and method of characterising volume.^28^

### Limitations

These data must be interpreted in the context of the study design. Misclassification of diagnosis or procedure could bias patient selection, although the direction of this bias is difficult to assess. Referral of more complex patients to specific hospitals or surgeons within the study cohort could explain between-hospital and between-surgeon variation, although that is unlikely given the non-overlapping referral patterns of most participating hospitals.^25^ Children may have had additional medical conditions that influenced timing of primary palate repair; we excluded children with complex chronic conditions,^22^ but we cannot eliminate the possibility of confounding by other medical conditions. We may have overestimated time to secondary surgery for censored individuals, as these children may have received secondary palate surgery at an institution other than their initial treating hospitals and this would not be in the dataset. Extent of variation among hospitals observed in figure 1b may not generalise to all children’s hospitals; the hospitals included in this figure were selected based on the large number of children undergoing palate repair at these hospitals during the observation period and may be outliers among all children’s hospitals.

## Conclusions

This large retrospective multicentre study demonstrated substantial variation in the hazard of secondary palate surgery for children with cleft lip and palate. Performing primary palate repair before 9 months of age substantially increases the hazard of secondary surgery, and choosing to perform palate repair after this age may be one approach to lowering rates of secondary surgery. Additional research is needed to identify other factors contributing to variation in palate surgery outcomes, along with testing of evidence-based interventions to decrease rates of secondary surgery. At present, these results suggest that cleft centres may be able to improve outcomes for their patients by adopting treatment practices from the best-performing centres. Performing cleft palate repair before 9 months of age may increase the risk of secondary procedures, unless the surgeon and centre have demonstrated successful long-terms outcomes.
