# Supplementary material for: Variation among cleft centres in the use of secondary surgery for children with cleft palate: a retrospective cohort study
Source: BMJ Paediatr Open. 2017 Aug 31;1(1):e000063. doi: 10.1136/bmjpo-2017-000063 (PMC5823530; doi:10.1136/bmjpo-2017-000063)
Supplement: Supplementary file 1 [file bmjpo-2017-000063supp001.pdf]

Table 3, Online Only. Adjusted hazard ratios for secondary palate surgery including year of primary palate repair as additional covariate.

| | Secondary Palate Surgery[a] | |
|---|---|---|
| Risk Factor | Hazard Ratio (95% CI) | *P* Value |
| Sex | | 0.71 |
| Male | 0.98 (0.87-1.10) | |
| Female | Reference | |
| Race | | 0.12 |
| White | Reference | |
| Black | 0.75 (0.58-0.97) | |
| Asian or Pacific Islander | 1.05 (0.80-1.37) | |
| American Indian | 1.14 (0.61-2.11) | |
| Other | 0.99 (0.82-1.19) | |
| Not specified | 1.27 (0.98-1.64) | |
| Median annual household income of ZIP code | | 0.15 |
| $33,525 or less (<1.5 FPL[b]) | Reference | |
| $33,526 - $44,700 (1.5-2 FPL) | 0.95 (0.82-1.10) | |
| $44,701 - $67,050 (2-3 FPL) | 0.84 (0.72-0.98) | |
| $67,051 or more (>3 FPL) | 0.91 (0.73-1.13) | |
| Age at primary palate repair | | <0.001 |
| <9 months[c] | | |
| At baseline | 7.00 (5.38-9.09) | |
| At 1 year after repair | 4.90 (3.57-6.72) | |
| At 5 years after repair | 1.18 (0.69-2.00) | |
| 9-15 months | 1.18 (0.96-1.46) | |
| 16-24 months | Reference | |
| Postoperative antibiotic use | | 0.01 |
| None | Reference | |
| Yes | 0.81 (0.69-0.95) | |
| Surgeon procedure volume (on day of repair) | | 0.13 |
| Low (<10 repairs in prior year) | Reference | |
| Medium (10-25) | 1.12 (0.95-1.33) | |
| High (>25) | 1.27 (1.01-1.61) | |
| Hospital procedure volume (on day of repair) | | 0.52 |
| Low (<25 repairs in prior year) | Reference | |
| Medium (25-50) | 0.96 (0.78-1.17) | |
| High (>50) | 0.86 (0.65-1.13) | |
| (cont'd) | | |

[a] Model assumes clustering of patients within surgeons and clustering of surgeons within hospitals; p<0.001 for likelihood-ratio tests of theta=0 for both surgeon and hospital
[b] FPL, US Federal Poverty Level for a family of four
[c] Age less than 9 months at primary repair is a time varying covariate, with baseline HR 7.00 (5.38-9.09) that decreases by 29.98% (26.10-33.65) each subsequent year

| | | |
|---|---|---|
| Length of stay after surgery | | 0.43 |
| ≤1 night | 1.05 (0.92-1.20) | |
| ≥2 nights | Reference | |
| Year of Primary Palate Repair | | <0.001 |
| 1997 | Reference | |
| 1998 | 1.12 (0.15- 8.42) | |
| 1999 | 1.22 (0.16- 9.20) | |
| 2000 | 1.35 (0.18-10.10) | |
| 2001 | 1.72 (0.23-12.89) | |
| 2002 | 1.59 (0.21-11.85) | |
| 2003 | 1.76 (0.24-13.12) | |
| 2004 | 1.00 (0.13-7.46) | |
| 2005 | 1.37 (0.18-10.26) | |
| 2006 | 1.29 (0.17-9.65) | |
| 2007 | 0.93 (0.12-7.03) | |
| 2008 | 0.97 (0.13-7.35) | |
| 2009 | 1.16 (0.15-8.78) | |
| 2010 | 1.13 (0.15-8.61) | |
| 2011 | 0.83 (0.11-6.39) | |
| 2012 | 1.13 (0.14-9.28) | |
| 2013 | 1.12 (0.15-8.42) | |
