# Supplementary material for: Variation among cleft centres in the use of secondary surgery for children with cleft palate: a retrospective cohort study
Source: BMJ Paediatr Open. 2017 Aug 31;1(1):e000063. doi: 10.1136/bmjpo-2017-000063 (PMC5823530; doi:10.1136/bmjpo-2017-000063)
Supplement: Supplementary file 3 [file bmjpo-2017-000063supp003.pdf]

Table 4, Online Only. Censoring time by age of primary palate repair. The duration of follow-up for children who did not undergo secondary surgery during the observation period, i.e. right-censored, is shown.

Age at primary palate repair	No.	No. without secondary surgery during observation period [right-censored] (%)	Censoring Time (yrs)		
			Mean	Median	
< 9 months	1164	638 (55%)	6.60	6.12	<0.001
9-15 months	3186	2413 (76%)	5.44	4.81	
16-24 months	589	467 (79%)	5.08	4.30	
