# Supplementary material for: Variation among cleft centres in the use of secondary surgery for children with cleft palate: a retrospective cohort study
Source: BMJ Paediatr Open. 2017 Aug 31;1(1):e000063. doi: 10.1136/bmjpo-2017-000063 (PMC5823530; doi:10.1136/bmjpo-2017-000063)
Supplement: Supplementary file 2 [file bmjpo-2017-000063supp002.pdf]

Figure 3, Online Only. Kaplan-Meier curves for time until secondary palate surgery by hospital, limited to hospitals with >175 patients undergoing palate repair during the observation period.

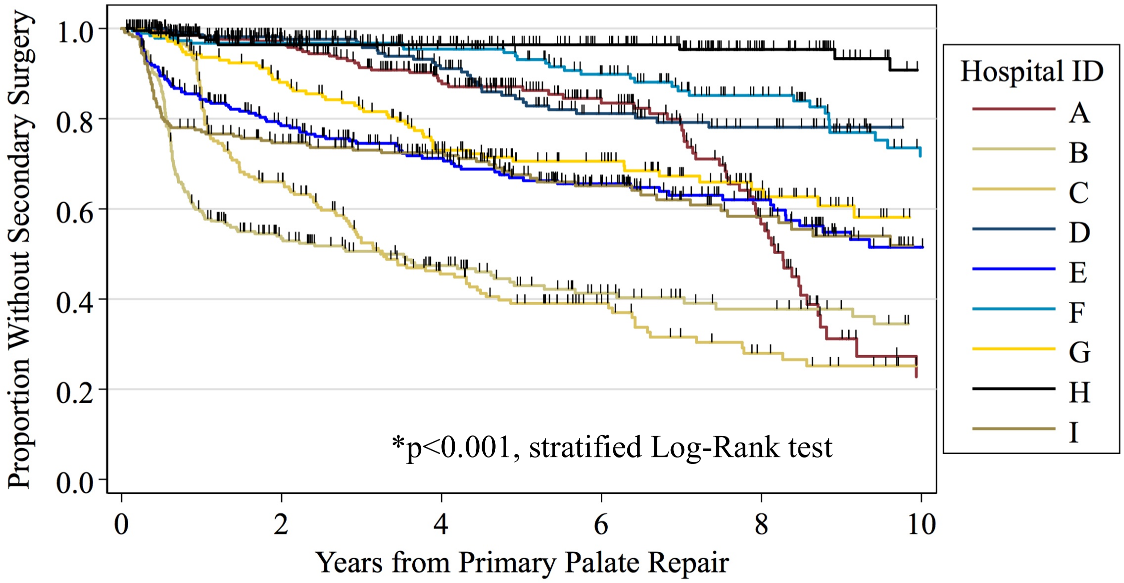

<u>N at risk</u>						
Hospital A	269	212	139	81	37	5
Hospital B	237	101	72	44	29	18
Hospital C	210	119	66	39	21	10
Hospital D	228	182	131	91	66	50
Hospital E	261	169	122	87	56	27
Hospital F	188	159	138	106	73	39
Hospital G	180	139	96	72	39	17
Hospital H	207	167	142	113	63	34
Hospital I	221	148	116	71	45	20
